# The Pathology and Treatment of the Herniæ of Children

**Published:** 1904-09-03

**Authors:** 


### Hospital Clinics.

### THE PATHOLOGY AND TREATMENT OF THE HERNIÆ OF CHILDREN.

There are two great factors in the production of hernia in children. (1) The congenital malformation in which the peritoneal cavity remains in communication with the tunica vaginalis of the testis. (2) An increase of abdominal pressure which actively produces the hernia and which is associated with gastro-enteritis or gastro-intestinal fermentation.

It is quite common to see multiple hernia in children following this second cause and indicating a general rather than a local origin for these protrusions. Mr. Edred Corner [1] calls attention to the great frequency with which a hydrocele in a child is found to communicate with the general peritoneal cavity. The fact that these hydroceles are hernia sacs, potentially so if they have never actually contained an abdominal viscus, demands hernial treatment. Again, as he points out, recent herniæ in adults frequently have congenital sacs, therefore the communicating strait is not sufficient alone to determine a hernia. The increased abdominal pressure is thus the more important factor. The larger number of hernial sacs operated on in childhood are acquired and unconnected with the tunica vaginalis.

That phimosis may be a cause of hernia cannot be denied, but Mr. Corner has rarely seen a case where this cause has been proved. Circumcision is always treatment of secondary importance as compared with diet and medicine. A small meatus urinarius is often associated with a tight prepuce and is the true cause of the straining during micturition. It is the persistence of the increased intra-abdominal pressure due to gastro-intestinal fermentation which gives rise to hernia rather than the sudden violent increments of pressure due to muscular effort and straining.

A truss may cure either by causing adhesions at the neck of the sac, or by preventing the descent of abdominal viscera, thus saving the sac from further distension and enabling natural growth to assist in, and perhaps perpetuate, the "cure." A skein of wool is soft and quite insufficient to cause these adhesions, but it aids in keeping viscera inside the abdomen. To the spring girdle-pad truss there are two main objections: First, the child is growing and the truss soon ceases to fit. In the second place, as the pressure will be exerted on the structure of the spermatic cord, as well as the neck of the sac, the testis is liable to suffer. Orchitis, atrophy, and hardening by fibrosis have been seen as the results of this truss. For the perfecting of the male characteristics, normal testicles, before the age of puberty, are essential. In studying people with imperfectly descended testes the conclusion is irresistible that the perfection of male characters are dependent upon the amount of perfect testicular tissue present. This being so, it seems impossible to cure a hernia which has a congenital sac by a truss that will cause no injury to the testis, unless the normal process of shutting the peritoneum from the tunica vaginalis is merely belated. Many herniæ in children disappear and are cured:—(1) Because the hernial sac is acquired, not congenital; (2) because the cause of raised intra-abdominal pressure for various reasons ceases to exist; (3) as the child grows concurrent increase in size and capacity of the body will lead to retraction of the acquired sac into the abdomen. In support of this last statement Mr. Corner mentions cases where he has operated on adults who gave a history of having had hernia during childhood, but in whom no trace of a congenital sac could be found at the operation.

A boy's testicle was in its proper place when a baby, later it became "imperfectly descended"; when operated on the testis was accompanied by a congenital hernial sac, suggesting that a congenital sac may also be occasionally drawn up into the abdomen as the result of the body's growth. Nevertheless, nothing short of operation is likely to cure a hernia in a congenital sac. Recumbency, grey powder, careful dieting, and a light soft truss may cure an acquired hernia in an infant. This treatment is good in each case till the age of four years; at or above that age all herniæ of children should be submitted to operation. A hernia which persists for some years in childhood causes permanent deformity of the internal abdominal ring, and it is not right to continue non-operative treatment, which will lead to a permanent deformation of the internal abdominal ring and the production of a hernia later in life. Four years is chosen as the age of election because the least in the way of operative interference is required, the internal ring will grow correctly afterwards, it is easier to perform the operation at this age than earlier, and the most radical operation that the patient's tissues permit can be performed then. The sac should be opened longitudinally and a purse-string suture carefully inserted, as high as practicable, and tied; then the operation may be completed by suturing the internal abdominal ring. The relationship between herniæ of children and adults is shown in the deformed ring, in the slight evidence we have that a congenital hydrocele ever loses its communication with the abdominal cavity, and in the large lax inguinal rings of some adults who, as children, suffered from the abdominal distension which has been emphasised as the chief cause of an acquired hernial sac.

The views put forward and derived from a study of inguinal hernia seem more likely to meet with acceptance in their application to the umbilical and ventral protrusions which exemplify all the points put forward in Mr. Corner's lecture.

When operating for the cure of umbilical hernia it is well to divide and suture the tissues in the middle line for some distance above and below the navel, remembering that in this form of rupture the recti are usually widely separated.

[1] Lancet, Aug. 20, 1904.

